# Evaluation of the impact of binocular versus monocular cataract surgery using Catquest-9SF: a randomized controlled trial

**DOI:** 10.5935/0004-2749.2023-0268

**Published:** 2024-09-16

**Authors:** Helmer Magalhães Antunes, Galton Carvalho Vasconcelos, Bruno Lovaglio Cançado Trindade

**Affiliations:** 1 Universidade Federal de Minas Gerais, Belo Horizonte, MG, Brazil; 2 Visão Instituto, Conselheiro Lafaiete, MG, Brazil; 3 Faculdade de Ciências Médicas de Minas Gerais, Belo Horizonte, MG, Brazil; 4 Instituto de Oftalmologia Cançado Trindade, Belo Horizonte, MG, Brazil

**Keywords:** Cataract, Cataract extraction, Quality of life, Treatment outcome, Visual acuity, Binocular vision, Stereopsis

## Abstract

**Purpose:**

This prospective, randomized, unmasked, clinical trial aimed to report the visual outcomes of cataract surgery on both eyes versus cataract surgery on one eye in Brazilian patients.

**Methods:**

This study included patients with bilateral cataracts and binocular visual acuity worse than or equal to 0.3 logarithm of the minimum angle of resolution. The patients were randomly assigned to undergo surgery on one (Control Group) or both eyes (one eye at a time; Intervention Group). Postoperatively, self-reported visual function using Catquest-9SF (primary outcome measure), binocular visual acuity, stereopsis, and ocular dominance (secondary outcome measures) were compared.

**Results:**

A total of 151 patients (77 and 148 eyes in the Control and Intervention Groups, respectively) completed the follow-up. Patients who underwent surgery on both eyes exhibited significantly better self-reported visual function (p=0.036) and stereopsis (p=0.026) than those who underwent surgery on one eye. Binocular visual acuity and ocular dominance did not affect the group comparisons.

**Conclusions:**

Surgery on both eyes resulted in significantly better self-reported visual function and stereopsis than surgery on one eye.

## INTRODUCTION

Cataract surgery on the second eye after the first eye has been surgically treated is common. It has become a standard clinical approach, subject to specific indications for each case. Due to the increasing number of cost-effectiveness studies in health care in recent years, cataract surgery on the second eye purportedly has limited clinical value^(1^,^2)^. Cataract surgery on one eye can improve daily visual function^(3^,^4)^, reduce the risk of falls, and decrease the incidence of fractures^(5^,^6)^ and other adverse events. However, surgery on the second eye can improve visual acuity, contrast sensitivity, and stereopsis^(6^,^7^,^8^,^9)^.

A recent meta-analysis revealed that few relevant randomized controlled trials measured the clinical effectiveness of surgery on the second eye^(10)^. All the evaluated studies had been conducted over 15 years prior; one then, even before the advent of phacoemulsification^(11)^, and another on a sample comprising only women^(6)^. All the studies were conducted in Europe, none in developing countries such as Brazil.

Visual acuity is used as a parameter for preoperative indications and evaluation of cataract surgery results. Other objective tests, such as visual field measurement, contrast sensitivity tests, and stereopsis, have been adopted to quantify the influence of media opacity on proper vision function. However, such tests do not fully measure the impact of visual dysfunction on individuals’ quality of life. Thus, outcome measures focused on symptoms, quality of life, convenience, and treatment costs have been frequently used^(12)^. Therefore, this study used an essential tool, namely, Catquest-9SF, to access the self-reported outcomes of patients undergoing cataract surgery. This tool was recently translated into Brazilian Portuguese and validated^(13)^.

## METHODS

### Study design and participants

This randomized controlled clinical trial with a parallel design was conducted in Conselheiro Lafaiete, Minas Gerais, Brazil. The study was approved by the research ethics committee of *Hospital das Clínicas da Universidade Federal de Minas Gerais* and was conducted in accordance with the principles of the Declaration of Helsinki.

**Figure 1:**
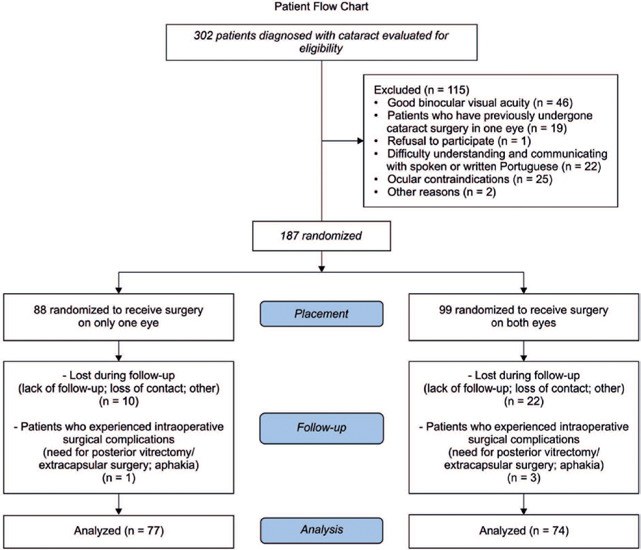
Patient flow chart.

Between May 2021 and June 2022, 302 patients who were indicated for surgery and on the public health system waiting list were called for evaluation. They provided informed consent after the nature and possible consequences of the study were explained to them. After voluntary acceptance, the patients were included if they met the following criteria: (1) the presence of binocular visual acuity worse or equal to 0.3 logarithm of the minimum angle of resolution (LogMAR) and (2) the need for cataract surgery on both eyes. Patients with cognitive difficulties or unable to understand spoken or written Portuguese, below 18 years old, with ocular comorbidities that may interfere with evaluation or follow-up (amblyopia, prior corneal surgery, clinically significant corneal dystrophies, severe corneal diseases, prior retinal detachment, and neuroophthalmologic disease), and requiring combined surgical procedures on one or both eyes were excluded (Figure 1).

### Sample size calculation

For the sample size calculation, data from similar previous studies that had determined that a 16% difference in vision acuity scores between the Intervention and Control Groups was sufficient and statistically validated was considered^(6)^. The estimated effect size was 0.32, and combined with a test power of 80% and 95% confidence interval, it yielded a sample size of 122 patients per group. An additional 20% was suggested to address probable losses and ensure a sufficient sample size throughout the study, thus indicating a sample size of 146 patients per group.

### Randomization and allocation

A simple randomization list based on a random sequence in a 1:1 ratio was computer-generated and pre-established by a statistics professional. After the initial enrollment, the principal investigator provided all the patients with an identification number for convenience, which was allocated sequentially.

The participants were divided into two groups:

**a) Intervention Group:** patients indicated for surgery on both eyes.
**b) Control Group:** patients indicated for surgery on only one eye.

If indicated, the patients allocated to the Control Group were invited to undergo surgery on the adelphic eye after the follow-up period.

### Monitoring and evaluation procedures

Patients in both groups had a similar follow-up schedule: initial evaluation (day 0) and evaluations on the 1st, 7th, and 30th postoperative days for each operated eye. On average, the total follow-up period was 2 months for the Control Group and 3 months for the Intervention Group. The 1-month difference between the groups generally represented the time between the first and second surgeries in the Intervention Group.

At the initial evaluation, in addition to mandatory ophthalmologic examinations and biometric calculations, all the enrolled patients underwent the following tests: (I) assessment of best-corrected binocular and monocular visual acuity (BCVA) using the Early Treatment of Diabetic Retinopathy Score, with the results converted to LogMAR, (II) cataract classification according to the Lens Opacities Classification System III^(14)^, (III) evaluation of ocular dominance using the hole-in-card test^(15)^, (IV) a stereopsis test using the Stereo Fly Test^(16)^, and (V) an evaluation of self-reported visual function using the translated and validated Brazilian Portuguese version of Catquest-9SF^(13)^

After the group-specific follow-up period, the participants were again subjected to tests I-V, except II. On average, the final evaluation occurred 1-2 months after the follow-up period. The first surgery was performed on the eye with the worst corrected vision (first option) or on the right eye (by convention) in cases with similar low visual acuity between the eyes.

### Self-reported visual function

Catquest-9SF is a tool for the self-assessment of cataract patients’ perception of their visual quality of life. This questionnaire, which comprises nine items, measures the visual problems the patient perceives in their daily life. Each item is scored on a scale that contains four numbered options theoretically, patients with high levels of visual impairment should choose the highest-scoring categories (3 or 4, representing greater difficulty/higher dissatisfaction), and those with low levels of impairment should choose the lowest-scoring categories (1 or 2). The questionnaire was translated into Brazilian Portuguese and revalidated in 2022^(13)^.

### Surgical procedure

A single experienced surgeon (the primary author) performed all the surgeries. The patients underwent phacoemulsification with foldable intraocular lens implantation. After the administration of mydriatic eye drops and anesthetics, a 2.4-mm corneal tunnel incision and a 1-mm side-port incision were made. Intracameral injection of lidocaine 1% was followed by injection with a viscoelastic substance to perform capsulorhexis and hydrodissection. Then, phacoemulsification was performed using a CataRhex 2 phacoemulsifier(Oertli Hafnerwisenstrasse, Berneck Switzerland) and a 0.9-mm curved tip of the device handpiece. Subsequently, moxifloxacin was injected intracamerally.

### Outcomes

The primary outcome of the study was the change in the self-perceived visual quality of life after surgery, as measured by the change in the Catquest-9SF score. The secondary outcomes included changes in binocular visual acuity patterns, stereopsis, and eye dominance patterns after surgery.

### Statistical analysis

Qualitative variables were expressed as absolute (n) and relative (%) frequency. The two groups were compared at baseline using a chi-squared test for qualitative variables and the Mann-Whitney U test for quantitative variables.

The results of Catquest-9SF were analyzed using generalized estimating equations, which enable the evaluation of the score variation between groups and in each group separately, considering the times at baseline and the end of follow-up. The same method was employed to assess the following visual functions: binocular visual acuity, stereopsis, and ocular dominance.

All statistical analyses were conducted using SPSS version 25 (IBM-Armonk, New York), with a significance level of 5%.

## RESULTS

### Sample characteristics

This study included 151 patients (77 belonging to the Control Group and 74 to the Intervention Group. The patients’ demographic and social characteristics, clinical comorbidities, and specific eye test results are shown in Table 1S, which showed no differences between the Groups.

**Supplemental table 1:** Baseline characteristics of the two study groups

	Surgery on one eye (n=77)	Surgery on both eyes (n=74)	p-value^a^
Age (Median – Q1–Q3)	71 (65.5–75)	69 (64–75)	0.693
Sex – Female (n – %)	48 (62.3)	50 (68.5)	0.428
Less than 5 years of formal schooling (n – %)	52 (69.3)	48 (64.9)	0.562
*Comorbidity (n* – *%)*			
Hypertension	40 (51.9)	49 (66.2)	0.075
Diabetes	28 (36.4)	19 (25.7)	0.156
Dyslipidemia	3 (3.9)	2 (2.7)	0.682
Hyperthyroidism	4 (5.2)	2 (2.7)	0.433
Heart disease	9 (11.7)	11 (14.9)	0.565
Lung disease	2 (2.6)	2 (2.7)	0.968
Arthritis	1 (1.3)	3 (4.1)	0.360
Spill	1 (1.3)	0 (0)	0.999
Falls and fractures	0 (0)	1 (1.4)	0.968
Other	14 (18.2)	20 (27)	0.193
Uncorrected binocular visual acuity (Median – Q1–Q3)	0.6 (0.5–1)	0.7 (0.5–1)	0.803
*Binocular visual acuity classification Without correction^b^ (n – %)*			
Slight (n–%)	24(21.2)	26 (35.1)	0.852
Moderate (n – %)	31 (40.3)	27 (36.5)	
Severe or worse (n – %)	22 (28.6)	21 (28.4)	
Binocular visual acuity with correction Median – Q1–Q3)	0.4 (0.4–0.65)	0.4 (0.3–0.6)	0.197
*Binocular visual acuity classification With correction (n – %)*			
Slight (n–%)	51 (66.2)	55 (74.3)	0.065
Moderate (n – %)	19 (24.7)	8 (10.8)	
Severe or worse (n – %)	7 (9.1)	11 (14.9)	
LOCS III – Nuclear Opacity OD (n – %)	69 (89.6)	69 (93.2)	-
LOCS III – Nuclear Opacity OD (Median – Q1–Q3)	2.5(1.75–2.9)	2.1 (1.3–2.9)	0.291
LOCS III – Nuclear Opacity OS (n – %)	69 (89.6)	67 (90.5)	-
LOCS III – Nuclear Opacity OS (Median – Q1–Q3)	2.3(1.5–2.9)	2.1 (1.4–2.8)	0.348
LOCS III – Cortical Cataract OD (n – %)	31 (40.3)	34 (45.9)	-
LOCS III – Cortical Cataract OD (Median – Q1–Q3)	1.9(1.3–2.8)	2.65(1.88–3.23)	0.075
LOCS III – Cortical Cataract OS (n – %)	33 (42.9)	36 (48.6)	-
LOCS III – Cortical Cataract OS (Median – Q1–Q3)	2.3(1.25–3.4)	2.8 (2.3–3.2)	0.179
LOCS III – Subcapsular cataract OD (n – %)	17 (22.1)	15 (20.3)	-
LOCS III – Subcapsular cataract OD (Median – Q1–Q3)	4.1 (2.75–5.25)	4.3 (3.4–5.9)	0.39
LOCS III – Subcapsular cataract OS (n – %)	14 (18.2%)	18 (24.3)	
LOCS III – Subcapsular cataract OS (Median – Q1–Q3)	2.8(1.43–4.63)	4.1 (1.28–5.03)	0.512
Ocular dominance for the right eye (n – %)	47 (61.8)	44 (62.9)	0.392
Stereopsis (n – %)			
Worse than 800	43 (57.3)	39 (52.7)	-
From 800 to 200	25 (33.3)	26 (35.1)	-
From 140 to 80	7 (9.3)	9 (12.2)	0.795
Catquest-9SF global score (Median – Q1–Q3)	21 (15.8–26)	21 (16.5–26)	0.923

a Chi-squared test for categorical variables and Mann-Whitney test for numerical variables.

b Classification according to the International Council of Ophthalmology^17^. LOCS = Lens Opacities Classification System III. OD = Right eye. OS = Left eye.

### Self-reported visual function results

The two groups were compared at baseline, and no difference was observed in the mean Catquest-9SF score (p=0.996), as expected. However, at the end of the study period, it was found that the intervention group had lower scores (lower difficulty/lower dissatisfaction) than the control group (p=0.036). When both groups were compared separately between the preoperative period and the end of the study period, there was a significant reduction in the total score obtained (p=0.0001). Therefore, it can be inferred that cataract surgery on one or both eyes led to a perceived improvement in both groups, although the difference was more significant in the intervention group (Table 1).

**Table 1:** Between- and intragroup comparisons of THE Catquest-9SF scores

	Baseline	End of follow-up	p-value ^a^
Monocular surgery	21 (15.5-26)	11 (10-13)	0.0001*
Binocular surgery	21 (16.8-25.5)	10.5 (10-12)	0.0001*
p-value	0.996	0.036*	

^a^Generalized equations were used for the intra- and intergroup analyses. Data are expressed as median and quartiles.

### Visual acuity results

Surgery on one or both eyes improved the corrected binocular acuity in both groups. However, the two groups did not significantly differ at the end of the follow-up period (Table 2).

**Table 2:** Inter- and intragroup comparisons of binocular visual acuity^b^ with the best correction

	Baseline	End of follow-up	p-value ^a^
Monocular surgery	0.4 (0.4-0.65)	0.2 (0.1-0.3)	0.000*
Binocular surgery	0.4 (0.3-0.6)	0.1 (0-0.3)	0.000*
p-value	0.817	0.752	

a Generalized equations were used for the intra- and intergroup analyses. Data are expressed as median and quartiles.

b Mean binocular visual acuity in the logarithm of the minimum angle of resolution.

### Stereopsis results

Over the study period, both groups exhibited improved stereopsis results (p=0.000). However, there was a significant reduction in the proportion of individuals with a result worse than 800 arc seconds (ArcSec) after surgery on one or both eyes.

The two groups were compared at the end of the study period, and a significant difference was observed between them (p=0.026). Overall, patients who underwent binocular surgery exhibited better fine depth discrimination than those who underwent monocular surgery (Table 3).

**Table 3:** Between- and intragroup comparisons of stereopsis n (%)

	Intervals *(ArcSec)*^a^	Baseline	End of follow-up	p-value
Monocular surgery	Worse than 800	43 (57.3)	15 (19.5)	0.000*
	From 800 to 200	25 (33.3)	26 (33.8)	
	From 140 to 80	7 (9.3)	35 (45.5)	
	From 60 to 40	0 (0)	1 (1.3)	
Binocular surgery	Worse than 800	39 (52.7)	8 (10.8)	0.000*
	From 800 to 200	26 (35.1)	18 (24.3)	
	From 140 to 80	9 (12.2)	42 (56.8)	
	From 60 to 40	0 (0)	6 (8.1)	
p-value		0.115	0.026*	

a Values in arc seconds (ArcSec).

### Ocular dominance results

No significant differences were observed when assessing the percentage of patients who underwent ocular dominance alternation between baseline and the end of the study period considering the same group (p=0.363) (Table 4).

**Table 4:** Intragroup dominance alternation

	Nonalternated dominance %	Alternated dominance %	p-value^a^
Monocular surgery	80.0	20.0	0.363
Binocular surgery	86.0	14.0	

a *Chi-squared test.*

## DISCUSSION

This study evaluated the differences in self-reported visual function, binocular visual acuity, stereopsis, and ocular dominance between patients who underwent monocular surgery and those who underwent binocular surgery. A significant improvement in self-reported visual function, measured using Catquest-9SF, in both groups was improved during the follow-up period. However, at the end of the follow-up period, more significant improvement was noted in those who underwent binocular surgery than in those who underwent monocular surgery. Similar studies have also demonstrated that patients who undergo monocular surgery experience significant improvement in self-reported visual function compared with those who have not yet had surgery on any eye; however, the self-perceived outcomes were significantly better in those who underwent binocular surgery than in those who underwent monocular surgery^(17^,^18^,^19)^.

Arsenault et al. compared the responses to Catquest-9SF among patients who underwent immediate sequential bilateral cataract surgery (surgery on both eyes on the same day), patients who underwent delayed sequential surgery (different days for the two eyes), and patients who underwent monocular surgery. Similar to the findings of this study, those who underwent binocular surgery had significantly lower scores on the questionnaire than those who underwent monocular surgery, indicating that binocular surgery improves self-reported visual function^(20)^.

Furthermore, binocular corrected visual acuity improved in both groups and did not differ between the groups at the end of the study period. As the eye with the poorest vision is the first choice in a surgical sequence, together with the fact that most patients in this study had mild visual impairment^(21)^ (BCVA 0.3-0.5 LogMAR), it can be inferred that cataract surgery, even if monocular, substantially improves binocular visual function.

The improvement in visual acuity in the groups in this study is relevant in that it corroborates the ability of Catquest-9SF to correctly measure self-reported visual function. Previous studies have also demonstrated a direct correlation between responses to Catquest-9SF and improved visual acuity following surgery^(13^,^22^,^23)^.

As noted at the end of the study period, the median visual acuity was obtained in the monocular and binocular groups (0.2 and 0.1 LogMAR, respectively). Previous studies have found that modern cataract surgery is successful in most patients with BCVA <0.2 LogMAR^(24^,^25)^. Despite being performed entirely with public funds in a tertiary hospital not specializing in ophthalmology, the surgeries in this study yielded excellent results, in no way inferior to the results obtained in private environments. Among the factors that contribute to these results are the improved access to basic supplies and equipment at affordable prices over recent decades, innovation of surgical techniques that have increased the degree of surgical reproducibility, experience of surgeons increasingly specializing in this subarea of ophthalmology, and incentives from the public sector in funding actions in this area supporting this surgeries.

As regards stereopsis, a significant improvement was observed in both groups during the segment, that is, although slight, there was also an important improvement in the group undergoing monocular surgery. Depth perception is aided by several “monocular cues” and cannot be explained by binocularity alone or the consequent cortical superposition of fields^(26^,^27)^. Thus, binocular vision is complex, and the contribution of monocular surgery to stereopsis is conclusive. Nevertheless, patients who underwent binocular surgery were undeniably better able to perceive fine depth than those who underwent monocular surgery, indicating that good binocular vision has advantages in the visual discrimination of space^(27)^.

Over half of the patients evaluated at baseline could not achieve stereoscopic results better than 800 ArcSec. This could be attributed to some specific factors of the sample, such as predominantly elderly individuals with physiological accommodative deficit, low cognitive capacity, and low visual acuity. In addition, several studies have reported that the prevalence of decreased stereopsis in the general population (even in those without known clinical abnormalities) ranges from 3% to over 30%^(26)^.

Finally, at the end of the follow-up period, few individuals achieved more refined stages of depth perception. Stereoacuity is strongly reduced when the visual acuity of one eye differs from that of the other, particularly at higher spatial frequencies. Likewise, when contrast is reduced more in one eye, there is greater impairment of stereoacuity compared to similar contrast reductions in both eyes^(26)^. These observations may be responsible for the low stereoscopic refinement rate.

The ocular dominance did not change in any of the groups. A recent study reported that the ocular dominance changed when the postoperative visual acuity of the nondominant eye improved relative to that of the dominant eye following surgery. However, if surgery on the contralateral eye is performed immediately after surgery on the dominant eye, the dominant eye returns to its initial state^(28)^. Such findings could not be demonstrated in this study. Variability is widely known to exist in the responses to repeated identical dominance tests. Approximately 40% of patients cannot indicate which eye is dominant^(29)^. Furthermore, there is no consensus regarding which of the several tests available is the most accurate^(15^,^30)^. These facts possibly influenced the findings of this study.

In recent years, substantial technical and technological improvements have been made in cataract surgery, which resulted in a highly reproducible and scalable procedure. However, cataract remains a significant public health concern, particularly in less developed and populous countries. Questions regarding the cost-effectiveness of cataract surgery must be addressed based on local realities. Despite the findings of this study, monocular surgery in situations where resources or opportunities are scarce is an excellent option and provides unquestionable functional gains.

This study has some limitations. First: this is a singlecenter study with a limited sample size conducted in a city in Minas Gerais. Second: self-reporting questionnaires, such as Catquest-9SF, are subjective as the patient’s overall experience can influence the results. Patients dissatisfied with the surgery, the attending physician, or even the lengthy process in the Brazilian public health system may report more vision-related difficulties than those who were satisfied. Third: postoperative residual refraction data, contrast evaluation using specific devices, and more advanced stereopsis tests may facilitate the generation of more relevant data for studies of this complexity.

In conclusion, our results indicate that self-reported visual function and stereopsis are significantly better in patients undergoing binocular surgery than in those undergoing monocular surgery. Binocular visual acuity and ocular dominance did not affect the results of the comparison between the groups. The visual function of both groups underwent positive changes throughout the segment, with no change in ocular dominance.
